# The influence of static and RF field heterogeneity on T1rho cardiovascular MRI

**DOI:** 10.1186/1532-429X-16-S1-P70

**Published:** 2014-01-16

**Authors:** Qiao Han, Yuchi Han, Robert C Gorman, Walter R Witschey

**Affiliations:** 1 Cardiology, University of Pennsylvania, Philadelphia, Pennsylvania, USA; 2 Surgery, University of Pennsylvania, Philadelphia, Pennsylvania, USA

## Background

Spin lock or T1rho MRI methods may be very useful for assessing myocardial disease. Recently, we reported that there is significant low field relaxation dispersion in myocardial and fibrotic scar tissue during in vivo T1rho MRI, contributing to contrast enhancement compared to spin echo (T2) MRI. However, we also observed that differences in magnetic field susceptibility between the heart and lung organs and poor RF penetration and field homogeneity in large patients and at 3 T contribute to T1rho quantification errors. Our goal was to determine the influence of static (B_0_) and RF (B_1_) heterogeneity on in vivo myocardial T1rho quantification.

## Methods

We performed simulations and experiments to explore how B_0 _and B_1 _field heterogeneity influences T1rho MRI. Tilted rotating frame (TRF) Bloch simulations were performed for a rotary-echo spin lock, varying field heterogeneity and spin lock duration (TSL = 2-50 ms, B_1 _= 60-140% nominal spin lock, and ΔB_0 _= -2-2 ppm at 1.5 T) with random Gaussian noise (σ = 1-20% of initial magnetization). Phantom experiments were performed using a homogeneous H_2_O cylinder (MnCl_2 _= 0.016%). In vivo experiments were performed in Yorkshire swine (n = 5). Simulated phantom and in vivo images were derived from B_0 _and B_1 _maps using the TRF Bloch simulations and were correlated with experimental images at the same spin lock duration.

## Results

**Figure 1:**
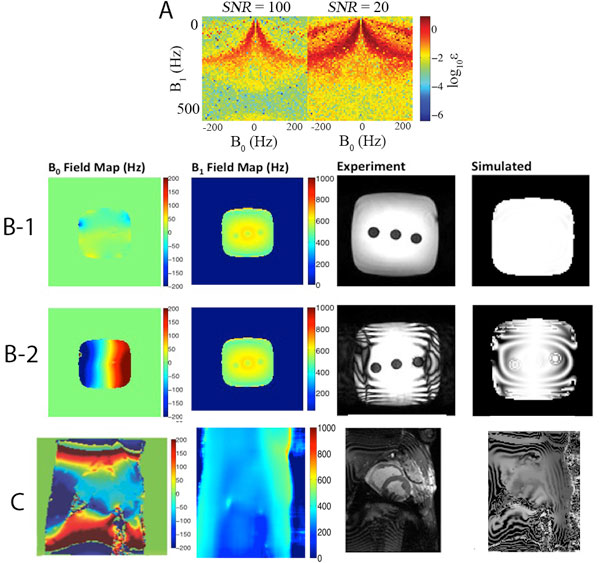
**The effect of field heterogeneity on T1rho relaxation times in the phantom and in vivo**. (A) Tilted rotating frame (TRF) Bloch simulations with random Gaussian noise. For the continuous wave 'rotary echo' spin lock pulse, measurement error was less than 1% for spin lock amplitudes greater than 400 Hz with minimal B_0 _field heterogeneity. Between ω1 = 200-400 Hz, this error could rise to 10%. (B) B_0 _and B_1 _field maps, experimental and simulated T1rho images in the phantom with no field gradient (1) and with 100 mT/m gradient (2). (C) B_0 _and B_1 _field maps, experimental and simulated T1rho images in vivo.

We investigated the effect of field heterogeneity on measured T1rho relaxation times in simulations, phantom experiments and in vivo. T1rho artifacts were characterized by banding artifacts resulting from incomplete longitudinal restoration of the T1rho-prepared magnetization, leading to RF field heterogeneity and rotation about the effective field during the spin lock pulse. These artifacts were most prominent at the LV posterolateral wall where significant static field heterogeneity (± 200 Hz) could result in nutation about an effective field oriented away from the idealized spin lock axis. In vivo results correlated well with simulations and phantoms experiments. Measured T1rho relaxation times in the three identical compartments were constant in the homogeneous B_0 _field and in the 50 mT/m field gradient in which the B_0 _field was less than 50 Hz off-resonance. Noticeable variations were observed at 100 Hz off-resonance (100 mT/m gradient). The B_0 _field in vivo is much less homogeneous than that for the phantom; therefore the T1rho images show much more complex patterns (Figure 1C). Mean B_0 _and B_1 _field over the left ventricle in the short axis view of the pig heart in vivo are in ranged -85.14 to 34.7 Hz and 244.30 to 361.87 Hz respectively with 95% confidence interval.

## Conclusions

Spin lock artifacts were the result of static field heterogeneity predominantly confined to the left ventricular posterolateral wall. Static field heterogeneity should be reduced to less than 10% of the spin lock field amplitude to minimize T1rho quantification errors.

## Funding

The authors gratefully acknowledge support from the National Institutes of Health through awards K99HL108157 and R01HL63904.

**Table 1:** 

Phantom					in vivo		
	**Gradient**	**Tube 1**	**Tube 2**	**Tube 3**		**Mean**	**Range**

B_0 _(Hz)	No gradient	16.89 ± 0.96	14.25 ± 0.80	8.68 ± 3.75	B_0 _(Hz)	-25.21	-85.14 - 34.7

	50 mT/s	−43.86 ± 5.58	14.18 ± 5.04	77.97 ± 7.63			

	100 mT/s	−103.43 ± 10.73	14.97 ± 10.21	148.84 ± 12.65			

B_1 _(Hz)	All gradient levels	546.38 ± 6.96	14.24 ± 0.80	541.50 ± 4.21	B_1 _(Hz)	303.01	244.29-361.87

T1Rho (ms)	No gradient	47.28 ± 2.92	47.25 ± 2.97	44.99 ± 3.06	T1Rho-Septum (ms)	55.55	50.13-60.97

	50 mT/s	43.02 ± 3.34	48.57 ± 2.92	45.57 ± 3.25	T1Rho-Anterior (ms)	55.27	46.29-64.24

	100 mT/s	59.40 ± 8.88	42.50 ± 4.02	63.26 ± 7.12	T1Rho-Posterolateral (ms)	60.60	45.64-75.57

